# A literature-derived dataset of migration barriers for quantifying ionic transport in battery materials

**DOI:** 10.1038/s41597-025-06196-x

**Published:** 2025-12-05

**Authors:** Reshma Devi, Avaneesh Balasubramanian, Keith T. Butler, Gopalakrishnan Sai Gautam

**Affiliations:** 1 Department of Materials Engineering, Indian Institute of Science, Bengaluru, 560012 Karnataka India; 2 Indian Institute of Science Education and Research, Pune, 411008 India; 3 Department of Chemistry, University College London, London, WC1E 6BT United Kingdom

**Keywords:** Batteries, Electronic structure

## Abstract

The rate performance of any electrode or solid electrolyte material used in a battery is critically dependent on the migration barrier (*E*_*m*_) governing the motion of the intercalant ion, which is a difficult-to-estimate quantity both experimentally and computationally. The foundation for constructing and validating accurate machine learning (ML) models that are capable of predicting *E*_*m*_, and hence accelerating the discovery of novel electrodes and solid electrolytes, lies in the availability of high-quality dataset(s) containing *E*_*m*_. Addressing this critical requirement, we present a comprehensive dataset comprising 621 distinct literature-reported *E*_*m*_ values calculated using density functional theory based nudged elastic band computations, across 443 compositions and 27 structural groups consisting of various compounds that have been explored as electrodes or solid electrolytes in batteries. Our dataset includes compositions corresponding to fully charged and/or discharged states of electrodes, with intermediate compositions incorporated in select instances. Crucially, for each compound, our dataset provides structural information, including the initial and final positions of the migrating ion, along with its corresponding *E*_*m*_ in easy-to-use .xlsx and JSON formats. We envision our dataset to be highly useful for the scientific community, facilitating the development of advanced ML models that can predict *E*_*m*_ precisely and accelerate materials discovery.

## Background & Summary

Ionic conductivity (*σ*) is one of the most important properties that is used to characterize materials used for electrochemical applications, such as a battery electrode or an electrolyte^1–4^. Typically, ionic conduction is a thermally activated process defined by the Nernst-Einstein equation as,

1 $$\sigma =\frac{{q}^{2}xD(x)}{{k}_{B}T}$$

where *q* and *x* are the charge and concentration of the intercalant (or the electroactive ion), respectively. *D*(*x*) is the diffusion coefficient of the intercalant that varies with *x*, *T* is the temperature and *k*_*B*_ is the Boltzmann constant. *D*(*x*) relates the diffusive flux (*J*) and the concentration gradient (∇ *x* of the intercalating species via the Fick’s first law (*J* = −*D*(*x*) ∇ *x*)^5^. Further, *D*(*x*) can be written as,

2 $$D(x)={D}_{J}(x)\theta (x)$$

*D*_*J*_ is the jump diffusion coefficient, which captures all the cross correlations among the individual atomic migrations and *θ* is the thermodynamic factor that captures the non-ideality of the solid solution (i.e., the interactions between the migrating ions and the host framework). *θ* is defined as $\theta =\frac{\partial (\mu /{k}_{B}T)}{\partial lnx}$, where *μ* is the chemical potential of the migrating ion. In solid electrodes and electrolytes, *x* is typically the site fraction of the migrating ion. For an ideal solid solution where each ionic hop has an identical hop frequency that is independent of the local concentration/configuration, *D*(*x*) becomes,

3 $$D=fg{a}^{2}\nu \exp \left(-\frac{{E}_{m}}{{k}_{B}T}\right)$$

*g* is the geometric factor that determines how the diffusion channels are connected, *f* is the correlation factor, *a* and *ν*, are the hop distance and vibrational prefactor, respectively, and *E*_*m*_ is the activation energy of migration. Ion transport within a crystalline lattice occurs through ionic migration events, where an ion moves from its original or interstitial site in a lattice to a neighboring vacant site, via a transition state. The migration process is influenced by the energy landscape encountered by the ion during its movement, with the *E*_*m*_ playing a crucial role in determining the ease of ionic mobility and, consequently, the material’s *σ*.

Extensive research has focused on enhancing ionic conductivity by minimizing *E*_*m*_, as this directly improves the rate capabilities of battery systems. Previous studies have shown the underlying host structure to play a vital role in influencing *D*, such as the presence of interconnected prismatic sites leading to improved Na^+^ mobility in P2-type layered structures^6^. In compositions like LiNiO_2_, Li off-stoichiometry leading to Ni^2+^ ions in the Li layers obstructing diffusion pathway can effect the Li-ion conductivity significantly^7^. Indeed, dopants that stabilize the layered structure, such as Ti^4+^ have been used to improve Na^+^ mobility^8^. In the case of phosphates, nuclear magnetic resonance (NMR)^9,10^ studies reveal that intercalant diffusivity is not governed by a single, uniform barrier but by a distribution of local energy barriers that are dictated by the arrangement of neighboring transition metal cations^11^. Additionally, subtle electrostatic distortions that screen electrostatic interactions between the intercalant and the anion framework have been shown to improve intercalant mobility in polyanionic structures^12^.

Galvanostatic intermittent titration technique (GITT)^13,14^ measurements in Mn and Fe rich disordered rocksalt structures have revealed the importance of Li-exccess compositions, particle size, and the underlying redox process as some of the important factors that affect the intercalant diffusivity^15–17^. Bonnick *et al*. illustrated the influence of poor electronic conductivity resulting in strong electrostatic interactions within thiospinel lattices (e.g., MgZr_2_S_4_) resulting in a reduction of ionic diffusivity^18^. In summary, various structural and chemical modifications have been explored across different types of intercalation compounds, including layered, spinel, olivine, polyanionic, and other frameworks, to enhance ionic conductivity^1,19–21^, with some approaches using targeted machine learning (ML) techniques as well^22–24^. However, developing universal optimization strategies across a wide range of intercalation systems remains challenging due to the interplay between structure, composition, and other factors besides the lack of a robust *E*_*m*_ dataset that spans a diverse range of materials.

In general, estimating diffusivities or *E*_*m*_ using experimental techniques like variable temperature NMR, GITT, and electrochemical impedance spectroscopy (EIS)^25,26^, are either experimentally challenging or resource intensive. This is due to the extremely short time scales (10^−12^ s) or small length scales ( ~ few Å) of the elementary process of ionic hopping, influence of surface and structural chemistry of electrodes/electrolytes on the measurement, variations in sample preparation and measurement conditions resulting in differences in interfacial formation, bulk stoichiometry and defects, and specific equipment requirements (e.g., the need for inert ion-blocking electrodes in EIS). Thus, experimental NMR/GITT/EIS data documenting *E*_*m*_ is unavailable for a wide range of materials.

Computational methodologies to estimate *E*_*m*_ have gained prominence, since calculated *E*_*m*_ can be used as a screening metric within high-throughput workflows before experimental validation. Computational techniques include empirical approaches such as bond valence sum (BVS^27,28^) analysis and nudged elastic band (NEB^29,30^) calculations (usually based on first principles simulations) or molecular dynamics (MD^31–33^). BVS analysis, though computationally swift, has accuracy limitations as it relies on an ionic bond model, making it more suitable for close-packed lattices with highly electronegative anions^34,35^. NEB calculations strive to estimate the migration barrier within a potential energy surface (PES) constructed by either density functional theory (DFT^36,37^) or interatomic potentials by modeling the ionic migration path using intermediate images that are connected by fictitious spring forces and subsequently relaxing the images to identify the saddle point that corresponds to the transition state. NEB calculations when performed in conjunction with DFT typically provide accurate *E*_*m*_. However, the DFT-NEB approach is computationally intensive for large systems (>100 atoms), and its accuracy/convergence can depend on the chosen exchange-correlation (XC) functional within DFT^38^. Classical MD (based on interatomic potentials) and ab-initio MD techniques can directly estimate *D*(*x*) at multiple *T*, thus yielding *E*_*m*_ from Equation (3), but are computationally demanding due to the time and length scales that need to be captured^39^. Note that ab-initio MD calculations are generally more accurate in estimating *E*_*m*_ or *D*(*x*) compared to classical MD due to the more accurate PES constructed by first principles.

Some strategies have been explored to reduce the computational costs and constraints, while retaining the accuracy, of both the DFT-NEB and ab-initio MD approaches. For example, the ‘pathfinder’ approach in conjunction with the ‘ApproxNEB’ scheme^40^ aims to reduce the computational constraints of DFT-NEB by mitigating convergence issues via selection of a ‘better’ initial migration path for calculation. However, the scheme still requires performing a full DFT-NEB calculation and is prone to the underlying constraints of the DFT-NEB approach. Another pathway is integrating ab-initio MD simulations with machine learned interatomic potentials (MLIPs), where the MLIPs can theoretically provide higher computational speeds with the accuracy comparable to DFT^41^. While several MLIP frameworks that are accurate remain chemistry-specific (i.e., there are high computational costs associated with training the MLIPs)^42–44^, the foundational or universal MLIPs^45–50^ have not been tested rigorously on *D*(*x*) or *E*_*m*_ predictions, yet. More importantly, we need datasets of *E*_*m*_ that are available over a wide-range of chemistries and structures to be able to test universal MLIPs in their utility in predicting *E*_*m*_ and/or build ML models that are tailored to accurately predict *E*_*m*_ that can be used for screening through materials.

In this work, we present a literature-based curated dataset of 621 DFT-NEB-derived *E*_*m*_ values across various compounds that have been studied as electrodes or solid electrolytes in lithium, sodium, potassium, and multivalent ion based battery systems. Our dataset includes fully charged and discharged states of electrode materials, with intermediate (non-stoichiometric) compositions considered in 30 cases. Additionally, we provide structural information for each compound, including the initial and final positions of the migrating ion, along with its corresponding energy barrier, which can be used in the construction of ML models that require structural inputs, such as graph-based models leveraging transfer learning^51^. Our dataset includes a total of 275 distinct entries contributed by 99 systems exhibiting multiple migration pathways. We envision our dataset to be a powerful resource for the scientific research and industrial communities, enabling the development of robust ML models and MLIPs that can eventually accelerate materials discovery for batteries and other applications.

## Methods

We conducted a thorough manual review of battery research articles published over the past two decades to compile computationally estimated *E*_*m*_ values. To ensure consistency and reliability, we focused exclusively on DFT-NEB calculated *E*_*m*_ values, as this method strikes a balance between accuracy and availability in the literature compared to other computational approaches. We note that the generalized gradient approximation (GGA^52^) is the most widely used XC functional among DFT-NEB calculations. Other functionals that are commonly used include Hubbard *U*^53^ corrected GGA (i.e., GGA+*U*^54^), local density approximation (LDA^55^), strongly constrained and appropriately normed (SCAN^56^) and its Hubbard *U*-corrected variant (SCAN+*U*^57^). Since GGA (and GGA+*U*) is computationally less intensive than SCAN/SCAN+*U* and gives reasonably accurate *E*_*m*_ estimations^38^, GGA (or GGA+*U*) is the preferred choice for DFT-NEB calculations. Our dataset reflects this preference, with 88.05% of the collected *E*_*m*_ values calculated using GGA, followed by GGA+*U* (7.27%), SCAN (3.07%) and LDA (1.61%). In cases where multiple XC functionals were used to calculate *E*_*m*_ within the same or different research articles, we prioritised GGA-calculated values to maintain consistency with the majority of the dataset. Note that for structures where the GGA/GGA+*U*-calculated *E*_*m*_ was not available, we included the *E*_*m*_ value as calculated with the different XC functional. Thus, the *E*_*m*_ barriers of non-GGA functionals have been used as is, and we have not done any re-calculation of such systems to ensure that all *E*_*m*_ have been calculated at the same level of theory. Given that *E*_*m*_ is primarily dependent on the local atomic environment, and temperature effects usually contribute to the pre-exponential factors in Equation (3), we do not expect entropic effects (such as configurational entropy) to play a major role in modifying *E*_*m*_ and hence do not include any temperature effects within our dataset. Nevertheless, if the presence of additional possible configurations in a structure unlocks new migration pathways, the *E*_*m*_ for such pathways can be calculated and the dataset be subsequently expanded.

We considered a research article for inclusion in our dataset only if it satisfied specific criteria ensuring the reliability and completeness of the reported DFT-NEB *E*_*m*_ values, i.e., we included studies that provided a comprehensive methodology for *E*_*m*_ calculations. Details on the methods we looked for in articles included the XC functional used, the number of intermediate images used to describe the migration pathway, and the supercell size employed in the NEB calculations. Articles that lacked the aforementioned details were excluded to maintain data consistency and accuracy. Structural information was another key criterion for selecting articles, where we only considered studies that provided explicit description of the parent structure(s), including the space group(s) and lattice parameters. In case of multiple articles reporting different *E*_*m*_ values for a given structure, we included the paper (and the corresponding *E*_*m*_) with the most details on the structure and the methods. If the structural parameters were missing and could not be retrieved from related works or structural repositories, we excluded the corresponding study from the dataset. For materials exhibiting multiple migration pathways, we ensured that the reported *E*_*m*_ were appropriately distinguished for each path via clear descriptions of the corresponding pathways. In cases where *E*_*m*_ values were not explicitly stated in text but were presented through minimum energy pathway (MEP) plots or other visualizations, only articles with clear, well-labeled plots featuring correct units and axis scales were selected to ensure accurate digital data extraction.

### Workflow

**Fig. 1:**
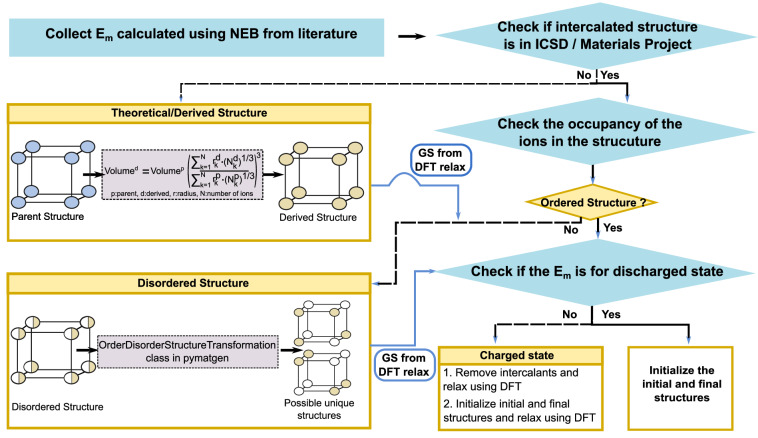
Flowchart illustrating the structural data generation process for each data point in the database. Rectangle and diamond symbols represent processes and decisions, respectively. GS refers to ground state. Relax refers to the structural relaxation calculation done with DFT.

Figure 1 presents an overview of the workflow for generating the structural information for our dataset. We analyzed the collected structural information for each datapoint, and ensured that the target structures were download from either the inorganic crystal structure database (ICSD^58^) or the materials project (MP^59^). If a target structure was available in both ICSD and MP, we downloaded the computationally relaxed structure from the MP. While DFT relaxation, such as the calculations performed within MP, can change the underlying lattice parameters and ionic positions compared to an experimentally refined crystal structure, we prioritized DFT-relaxed structures so that our dataset is internally consistent with DFT-calculated *E*_*m*_. For all electrode materials, where possible, we downloaded the structure of the discharged composition (i.e., structures with high concentrations of intercalant ions, relevant for electrode materials) preferably over the charged composition.

If the target structure was not available in either ICSD or MP, we searched for a parent structure that shared the same space group, migrating ion concentration, and site occupancies, but containing ions that are different from the target structure in both ICSD and MP. The parent structure was then used as a template, where we substituted the occupant ions with the target ions and used the reference lattice scaling (RLS^60^) scheme, as implemented by the RLSVolumePredictor class in the pymatgen package^61^, to obtain a target structure with scaled lattice parameters and the right composition. If target structures were available but contained sites with partial occupancies, we enumerated all possible symmetrically unique configurations that satisfied the target stoichiometry of the reported structure by using the OrderDisorderStructureTransformation class in pymatgen. For all the RLS-generated and/or enumerated structures that we obtained, we performed structure relaxations with DFT to obtain the ground state (i.e., lowest energy) configuration.

For all structure relaxations with DFT, we used the GGA XC functional as available in the Vienna ab initio simulation package (VASP, version 6.1.2)^62,63^. The effects of core electrons were described via projector augmented wave potentials^64^. We relaxed the lattice vectors, cell volume, and ionic positions of all input structures, without preserving any symmetry, until the total energy and atomic forces were below 0.01 meV and |0.03| eV/Å, respectively. We used a Γ-centred *k*-point mesh with a density of at least 48 *k*-points per Å^−1^ for sampling the irreducible Brillouin zone, a kinetic energy cutoff of 520 eV for describing the plane-wave basis set, and a Gaussian smearing of width 0.05 eV to intergrate the Fermi surface. For select structures that exhibited convergence difficulties with GGA, we performed the structural relaxations with GGA+*U*, utilizing the optimized values reported in Ref. ^54^ All our structures are compatible with the structure relaxation calculation settings of MP (version 2020).

Upon obtaining the ground states for all systems in our dataset, we reviewed the migration path information reported in the corresponding research articles and initialized the initial and final configurations of the migration path within each structure. Note that the initial configuration represents the migrating ion occupying the starting site while leaving the destination site vacant, while the final configuration depicts the inverse arrangement. Thus, we have assumed that all migration events for all structures considered in our dataset occur via a vacancy-based mechanism and not an interstitial-based mechanism. All the solid electrolyte materials in our dataset are stoichiometric and ordered compositions, hence we treated them as equivalent to discharged compositions of electrode materials in our workflow.

For electrode structures where the *E*_*m*_ was reported for a charged composition (i.e., the dilute ion limit), we removed the intercalant ions from the ground state discharged structure and subsequently relaxed the structure using DFT. The initial and final configurations were then defined in this relaxed charged structure and were DFT-relaxed again to obtain their true ground state descriptions. In the case of intermediate intercalant compositions, all symmetrically distinct positional configurations corresponding to the composition were enumerated, followed by DFT relaxation to identify the ground state, and the corresponding pathway initializations were carried out in the ground state. For generating all initial and final configurations, we selected appropriate supercell sizes to ensure that the migrating ion does not experience spurious interactions by maintaining a minimum distance of at least 8 Å with its periodic images. In structures with large unit cells, such as NaSICONs (sodium superionic conductors), weberites, and oxyfluorides, we did not generate supercells to reduce computational costs.

## Data Records

The dataset is available at our GitHub^65^ and Zenodo^66^ repositories, with this section describing the availability and content of the data. We report computationally calculated *E*_*m*_ of 621 systems that have been explored as battery materials along with their structural information for each possible ionic migration event. The data can be easily downloaded in the form of a JSON file from our GitHub^65^ or Zenodo^66^ repositories.

### File format

Each datapoint in the dataset is associated with specific tags that provide essential information, as summarized in Table 1. For each datapoint, we include its composition, crystal class, space group, unique system identifier, and a JSON ID that differentiates each datapoint within the database and allows easier access to different migration paths within a given structure. We assign the system name for each datapoint using a standardized format: *reduced_chemical_formula* + ‘_’ + *path_number*. For instance, MgCoSiO_4_ has two possible migration paths for Mg^2+^ diffusion, so we represent each path as *MgCoSiO4_1* and *MgCoSiO4_2*. However, if a composition has only one active migration path, it is identified using only the reduced chemical formula. Parentheses and subscripts are omitted in the system name generation.

**Table 1:** Description of each tag associated with the datapoints in the *E*_*m*_ dataset.

S.No	Tags	Description
1	jid	Unique JSON ID
2	structure_ini	Initial configuration of the migration path
3	structure_fin	Final configuration of the migration path
4	target	Reported *E*_*m*_
5	formula	Chemical composition
6	crystal_class	The crystal class
7	sys_name	A unique system name
8	space_group	Space group
9	XC	XC functional used for the NEB calculation in literature
10	bibtex	Citation of the research article in ‘bibtex’ format
11	calc_metadata	Dictionary containing DFT calculation details with four keys: SOFTWARE_INFO (software and version), PP_INFO (pseudopotential and valence electrons detail), NEB_CONVERGENCE (NEB thresholds), RELAX_CONVERGENCE (structure relaxation thresholds), and OTHER_DETAILS_SPECIFIED (notes on energy cutoff, *k*-points, *U* parameters, and spacing between the periodic images).

When multiple polymorphs of the same composition exist, the system name is modified to include the space group: *reduced_chemical_formula* + ‘_’ +* space_group* + ‘_’ + *path_number*. Additionally, for layered structures where both monovalent and divalent hops are considered^67^, we treated both hops as distinct migration paths. Charged state structures are labeled using the format: *charged_state_reduced_chemical_formula* + *_* + *intercalant*, allowing clear differentiation from the discharged state configurations. In order to be compatible with the notations used in the original papers, certain prefixes, such as O3:O3-layered structure, O:olivines, M:maricite, d:*δ*, e:*ϵ*, g:*γ*, b:*β*, a:*α*, and a1:*α*1, were added to the corresponding system names to ease the identification. We did not modify our notation for intermediate compositions as compared to the discharged compositions.

The dataset provides structural information for both the initial and final configurations of each migration path in the ‘POSCAR’ format, which is compatible with pymatgen and the atomic simulation environment (ASE^68^) packages, enabling easy conversion into other structural representations. Each datapoint also includes a “bibtex” tag, which contains the citation details of the article from which the *E*_*m*_ values and the migration path information were sourced. Additionally, the XC functional used to calculate the *E*_*m*_ in the respective study is provided under the “XC” tag for reference. We acknowledge that other calculation settings, such as the potential used for describing core electrons, the mesh density and scheme used for sampling *k*-points in the irreducible Brillouin zone and integrating the Fermi surface, and convergence criteria employed on energies and forces, can influence the calculated *E*_*m*_ as well, albeit to a lesser extent compared to the XC functional. To capture such DFT calculation settings in the dataset, we provide the metadata for both structural relaxations and NEB calculations, as detailed in the respective articles, in the form of a dictionary under the tag “calc_metadata”. If any specific information is not available, we populate the corresponding entry with a ‘NaN’.

## Data Overview

**Fig. 2:**
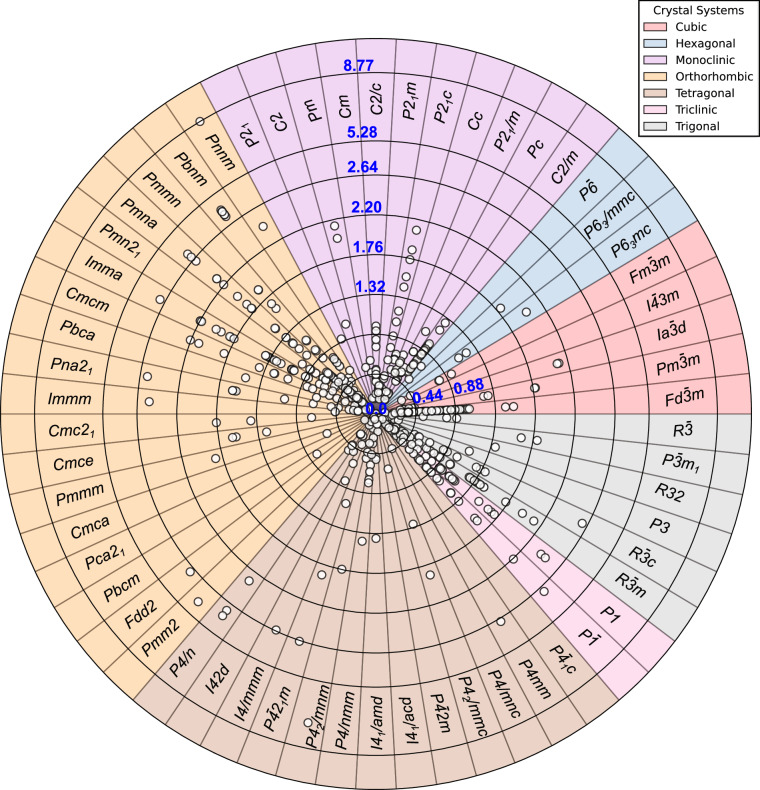
Contour plot illustrating the distribution of the *E*_*m*_ dataset over different space groups from each of the seven crystal systems. Individual colored sectors represent individual crystal systems, with space groups indicated by text notations. White circles indicate invidual data points. Concentric circles correspond to different E_*m*_ values (in eV), as highlighted by the blue text notations.

The distribution of *E*_*m*_ across the seven crystal systems, and the corresponding space groups within each crystal system, in our dataset is illustrated as a contour plot in Fig. 2. Each crystal system is visually distinguished using color-coded sectors in Fig. 2, with the solid concentric rings representing different *E*_*m*_ values (in eV). Overall, our dataset includes *E*_*m*_ values spanning 58 space groups and ranging from 0.03 eV to 8.77 eV. Out of the 621 datapoints, 528 are electrodes and 91 are electrolytes. The lowest *E*_*m*_ (0.03 eV) corresponds to charged-LiTiO_2_ (*P42/mnm*), while the highest (8.77 eV) is observed in discharged-LiRuO_2_ (*Pnnm)*, respectively. Notably, both LiTiO_2_ and LiRuO_2_ adopt the rutile-type structure and consequently exhibit the widest range of *E*_*m*_ values in the dataset. In contrast, space groups $Ia\overline{3}d$ and *I41/acd*, which contain two and three datapoints, respectively, show the narrowest range of *E*_*m*_ values.

The space group $Fd\overline{3}m$, corresponding to cubic spinels, has the highest number of datapoints, contributing 94 entries to the dataset. It is followed by the *Pmna* space group (72 datapoints), belonging to the orthorhombic system, and the *P1* space group (39 datapoints) from the triclinic system. Additionally, 15 space groups contribute only a single datapoint each. Among the different crystal systems, orthorhombic accounts for the highest (205) number of datapoints, whereas hexagonal (6) contributes the least. The dataset exhibits a mean *E*_*m*_ value of 0.848 eV with a standard deviation of 0.824 eV, indicating a highly skewed distribution. A majority (73.4%) of the *E*_*m*_ values are below 1 eV, while 19.4% fall within the 1-2 eV range, and 7.2% exceed 2 eV. 71.4% and 23.6% of the entries are contributed by discharged (high intercalant content) and charged (low intercalant content) state structures respectively, corresponding to 106 distinct charged and discharged pairs. Intermediate intercalant compositions correspond to 5% of the dateset.

**Fig. 3:**
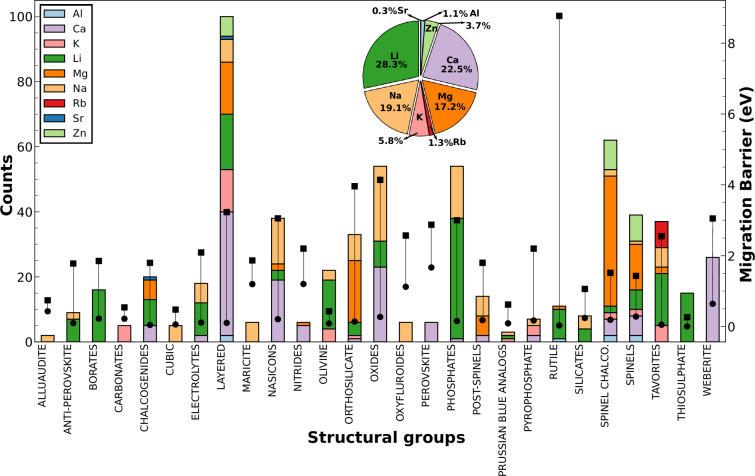
Illustration of the *E*_*m*_ distribution within each of the different structural groups. The *y* axis on the left and right represent the number of datapoints and *E*_*m*_ values (in eV), respectively. Stacked bar charts correspond to the counts within each structural group, with the colors indicating the split across various intercalants. The black vertical lines represent the range of *E*_*m*_ values for a given structural group with the squares and circles representing the maxima and minima, respectively. The inset shows a pie-chart with the contributions from each intercalant (i.e., Al, Ca, K, Li, Mg, Na, Rb, Sr, and Zn, as represented by the different colors) to the total dataset.

Figure 3 presents a bar chart that illustrates the number of datapoints and the range of *E*_*m*_ values across 27 different structural groups (e.g., spinels, olivines, NaSICONs, etc.). Each bar is stacked to represent contributions from nine different intercalating ions, with the stack length indicating the number of datapoints associated with each ion. The inset pie chart provides a breakdown of the percentage contribution of each intercalating ion to the overall dataset. Additionally, the solid square and circle markers, connected by a vertical line, denote the maximum and minimum *E*_*m*_ values observed within each structural group, thus representing the range of *E*_*m*_.

Lithium-based intercalant compounds constitute 28.27% of the dataset, making Li the most prevalent intercalating ion, which is expected given the extensive research done on Li-based electrodes and solid electrolytes. Li is followed by calcium (Ca), sodium (Na), and magnesium (Mg) in terms of contribution. The least represented intercalants are strontium (Sr), aluminum (Al), and rubidium (Rb), with only 2, 7, and 8 datapoints, respectively. 17 out of the 27 structural groups include compounds intercalating Li, whereas Ca-based compounds are primarily found in layered structures, NaSICONs, oxides, and weberites. Na-based compounds are more widely distributed than Li, appearing in 19 of the 27 structural groups, while Mg-based compounds are predominantly found in spinel chalcogenides, layered structures, and orthosilicates. Layered structures contribute the highest number of datapoints, with 98 entries, followed by spinel chalcogenides, phosphates, and oxides. Other structural groups, such as alluaudites, Prussian blue analogs, and carbonates, are also represented but in significantly smaller numbers. The highest and lowest *E*_*m*_ ranges among the structural groups are observed in rutiles and thiosulphates, respectively.

## Technical Validation

A benchmarking of DFT-NEB E_*m*_ (using different XC functionals) against experimentally reported values has been performed in our previous work^38^. To estimate *E*_*m*_, we fully relaxed the endpoint geometries representing the initial and final states of migration using DFT. Subsequently, the MEP was initialized by linearly interpolating both atomic positions and lattice vectors to create seven intermediate images between the endpoints, with a spring force constant of 5 eV/Å^2^ maintained between adjacent images. The images constituting the NEB were optimized along the reaction coordinate using the limited-memory Broyden-Fletcher-Goldfarb-Shanno (L-BFGS)^69^ method until the force component perpendicular to the elastic band fell below |0.05| eV/Å. The total energy of each image was also converged to within 0.01 meV. All *E*_*m*_ values were determined assuming a vacancy-mediated mechanism in the dilute-vacancy limit.

On comparing the calculated *E*_*m*_ against experimental values, we observed the SCAN functional to exhibit a higher accuracy on an average relative to other XC functionals. Albeit the higher accuracy of SCAN is counterbalanced by increased computational expense and potential convergence problems. Also, we found GGA to be a suitable alternative for quick and qualitative *E*_*m*_ predictions.

Approximately 15.8% of the dataset has been calculated from scratch using the GGA or SCAN XC functional (depending on the computational feasibility for the respective system), using the above described methodology and have also been reported in other works^38,70–74^. The remaining 84.2% of the dataset has been collected from various literature sources that report DFT-NEB methodologies similar to the above description. In certain cases, articles that used fewer number of images (3 or 5) were also considered if a fully convereged MEP was reported.

## Usage Notes

We present a literature-curated DFT-NEB-calculated dataset comprising 621 distinct *E*_*m*_ values over 443 chemistries and 27 distinct structural groups, spanning a diverse set of electrode and solid electrolyte materials studied for battery applications. This dataset, which includes structural information and calculated *E*_*m*_ values for each system, is provided in both .xlsx and JSON formats for easier and direct data extraction within our GitHub^65^ repository. The JSON version of the dataset is also available on Zenodo at the DoI^66^. The following code snippet demonstrates the conversion of structural data from the dataset into pymatgen and ASE objects for subsequent analyses. Our dataset can be effectively utilized to construct ML models for *E*_*m*_ estimation, using either structural, compositional, or combined inputs. The snippet illustrates that the dataset can be imported directly into a pandas DataFrame, enabling its use in further machine learning model development, using libraries such as ‘scikit-learn’ and ‘pytorch’.

Conversion of JARVIS structure to pymatgen and ASE objects. 

We are hopeful that the dataset will be expanded in the near future with calculated *E*_*m*_ contributions from the scientific community. We have provided instructions on contributing to the dataset in our Zenodo repository, which will be used for maintaining version-control as well. With further expansion, the dataset can be employed to develop accurate machine learning models capable of replacing on-the-fly DFT-NEB estimations of *E*_*m*_, which would significantly accelerate kinetic Monte Carlo (kMC) simulations^75^. In turn, faster kMC simulations can bridge the gap between the high accuracy of DFT and the long timescales accessible by kMC, ultimately providing a powerful tool for quantifying ion transport dynamics. Additionally, the dataset will be suitable for fine-tuning pre-trained foundational models and for benchmarking the performance of various universal MLIPs on *E*_*m*_ prediction tasks.

## Data Availability

The dataset developed as part of this work is available freely online at our GitHub (https://github.com/sai-mat-group/migration-barrier-dataset) repository and on Zenodo at the, 10.5281/zenodo.17240095.

## Code availability

There are no specific codes or scripts developed in this work. Licenses for using the VASP code, which was employed for the DFT calculations done in this work, are available at https://vasp.at/. The pymatgen package can be downloaded freely from https://pymatgen.org/.
